# Levels of miR-126 and miR-218 are elevated in ductal carcinoma *in situ* (DCIS) and inhibit malignant potential of DCIS derived cells

**DOI:** 10.18632/oncotarget.25261

**Published:** 2018-05-04

**Authors:** Stefano Volinia, Valeria Bertagnolo, Silvia Grassilli, Federica Brugnoli, Marco Manfrini, Marco Galasso, Cristian Scatena, Chiara Maria Mazzanti, Francesca Lessi, Giuseppe Naccarato, Adelaide Caligo, Enzo Bianchini, Quirino Piubello, Enrico Orvieto, Massimo Rugge, Cristina Natali, Domenico Reale, Andrea Vecchione, Sarah Warner, Carlo Maria Croce, Silvano Capitani

**Affiliations:** ^1^ Department of Morphology, Surgery and Experimental Medicine, University of Ferrara, Ferrara 44121, Italy; ^2^ LTTA Centre, University of Ferrara, Ferrara 44121, Italy; ^3^ Department of Translational Research and New Technologies in Medicine and Surgery, University of Pisa, Pisa 56126, Italy; ^4^ Pisa Science Foundation, Pisa 56121, Italy; ^5^ Pathology Division, S. Anna University Hospital, Ferrara 44124, Italy; ^6^ Department of Diagnostic and Pathology, Azienda Ospedaliera Universitaria Integrata di Verona, Verona 37126, Italy; ^7^ Department of Medicine DIMED, University of Padova, Padova 35121, Italy; ^8^ Pathology Division, Santa Maria della Misericordia Hospital, Rovigo 45100, Italy; ^9^ Department of Pathology, St. Andrea University Hospital, University of Rome, La Sapienza, Rome 00185, Italy; ^10^ Comprehensive Cancer Center, Ohio State University, Columbus, OH 43210, USA

**Keywords:** DCIS, breast tumor progression, EMT, breast cancer, over-diagnosis

## Abstract

A substantial number of ductal carcinoma *in situ* (DCIS) detected by mammography never progress to invasive ductal carcinoma (IDC) and current approaches fail to identify low-risk patients not at need of adjuvant therapies. We aimed to identify the key miRNAs protecting DCIS from malignant evolution, that may constitute markers for non-invasive lesions.

We studied 100 archived DCIS samples, including pure DCIS, DCIS with adjacent IDC and pure DCIS from patients with subsequent IDC in contralateral breast or no recurrence. A DCIS derived cell line was used for molecular and cellular studies.

A genome wide study revealed that pure DCIS has higher miR-126 and miR-218 expression than DCIS with adjacent IDC lesions or than IDC. The down-regulation of miR-126 and miR-218 promoted invasiveness *in vitro* and, in patients with pure DCIS, was associated with later onset of IDC. Survival studies of independent cohorts indicated that both miRNAs play a protective role in IDC. The clinical findings are in agreement with the miRNAs’ roles in cell adhesion, differentiation and proliferation.

We propose that miR-126 and miR-218 have a protective role in DCIS and represent novel biomarkers for the risk assessment in women with early detection of breast cancer.

## INTRODUCTION

Breast cancer (BC) is characterized by several histological and genetic distinctive features, which lead to substantial differences in treatment and clinical outcomes [1, 2]. After the advent of screening mammography, the proportion of detected early carcinomas increased substantially and only 20% of them were expected to progress, which implied a high number of over-diagnosed lesions [3]. Well-established models of breast cancer evolution propose an apparently continuous but non-obligatory progression through a series of increasingly abnormal stages, including the carcinoma *in situ*. Ductal carcinoma *in situ* (DCIS) represents 20–25% of newly diagnosed BC in industrialized countries and up to 40% of DCIS lesions progress to invasive carcinoma (IDC) if untreated [4]. Similarly to IDC, DCIS is a heterogeneous group of breast lesions and the potential for progression to invasive carcinoma varies among the molecular subtypes [5–9]. The evaluation of clinical biomarkers in adjacent DCIS and IDC allowed determining that different degrees of aggressiveness characterize DCIS lesions with high heterogeneity in the same patient. An intra-tumor genetic heterogeneity was also observed in adjacent DCIS and IDC, indicating that tumor cells with specific genetic and/or epigenetic variants may be selected during progression [10–12]. Definitely and at variance with IDC, neither traditional classification systems, nor molecular characterizations can reliably predict whether DCIS will progress. In addition, the intracellular pathways that control progression to IDC are still largely unknown, making it difficult to identify either robust biomarkers or therapeutic targets for DCIS.

Since present approaches do not allow accurate risk stratification, low-risk patients are particularly poorly defined in terms of need for adjuvant therapies, with subsequent generation of short-term and/or long-term effects affecting all-cause mortality. It is then clearly emerging that efficient diagnostic assays predicting the risk of recurrence and/or progression of each DCIS will play a crucial role in the selection of individual therapy [13].

MicroRNAs (miRNAs), conserved single-stranded RNA molecules, are non-coding RNAs that post-transcriptionally regulate gene expression. MiRNAs control many cancer hallmarks such as proliferation, growth suppression, cell death, angiogenesis, invasion and metastasis, but also genome instability and inflammation [14]. Although several studies have investigated miRNAs in many aspects of breast cancer [15, 16], lesser attention has been devoted to the progression of DCIS to IDC. In addition, due to tumor heterogeneity, large variation exists among the few available miRNA profiles of DCIS, performed in very small cohorts and not investigated, however, for their functional properties [12, 17, 18].

Our work was aimed to identify miRNAs with an effective role in the path/s from *in situ* to invasive BC. This study includes formalin-fixed tumor samples from 4 DCIS patient groups: a test cohort of pure DCIS and three validation cohorts formed by pure DCIS, DCIS with adjacent IDC, pure DCIS from patients with, and without subsequent IDC in contralateral breast.

## RESULTS

### miR-126 and miR-218 are elevated in pure DCIS

**Figure 1:**
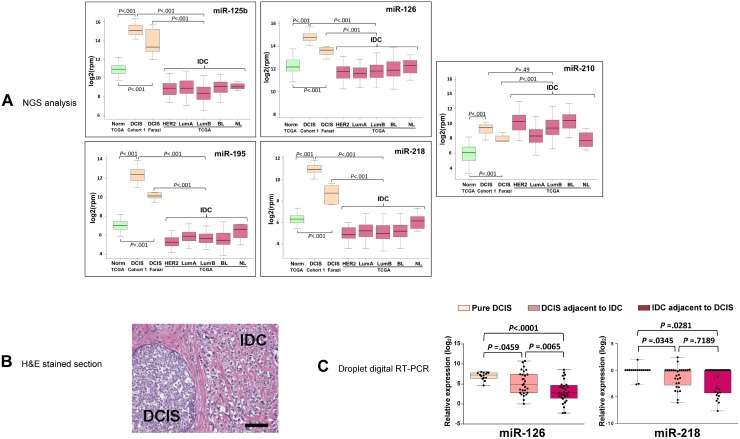
The expression of miR-126 and miR-218 is elevated in pure DCIS but not in DCIS adjacent to IDC (**A**) Box plots showing the difference in expression of miR-125b, miR-126, miR-195, miR-218 and miR-210 in DCIS (Cohort 1, *n* = 30), in the Farazi cohort (DCIS Farazi, *n* = 7) and in the invasive ductal carcinoma subtypes of the TCGA cohort (Norm: Normal, *n* = 100; HER2: HER2 enriched, *n* = 56; LumA: Luminal A, *n* = 226; LumB: Luminal B, *n* = 120; BL: Basal-like, *n* = 89; NL: Normal-like, *n* = 8). Expression levels of miRNAs (reads per million, rpm) were log2 transformed. (**B**) Representative haematoxylin and eosin (H&E) stained section of breast tumors containing adjacent DCIS and IDC lesions (Cohort 3, *n* = 30). Bar = 100 μm. (**C**) Droplet Digital RT-PCR analysis of miR-126 and miR-218 in micro-dissected sections of pure DCIS (Cohort 2, *n* = 17) and adjacent DCIS and IDC (Cohort 3). The expression levels of each miRNA are indicated as log2 ratio over its cohort median level.

To gain new insights into the miRNomics of DCIS, we profiled a collection of 30 pure *in situ* breast tumors (Cohort 1) by performing RNAseq on small RNA. The expression of 1222 different mature miRNAs was measured across all DCIS samples. The miRNA-Seq profiles were compared to those of other DCISs from the Farazi's cohort [17] and of IDCs from the TCGA cohort. miR-125b, miR-126, miR-218 and miR-195 were over-expressed in DCIS vs. IDC (*P* < 0.001) and their over-expression was confirmed in the small Farazi DCIS cohort (*P* < 0.001) (Figure 1A). Among these miRNAs we successfully cross-validated miR-126 and miR-218 when using the samples from the Norway study by Sorlie and coworkers [12] (Supplementary Figure 1). miR-210 was previously reported to be associated with DCIS malignant transition [18] and this study confirmed its over-expression in DCIS with respect to normal tissues (*P* < 0.001), but not to IDC (Figure 1A).

We then studied the levels of validated miR-126 and miR-218 in patients with DCIS adjacent to invasive carcinoma (Figure 1B). Digital RT-PCR after laser capture micro-dissection revealed that the DCIS samples with adjacent IDC (Cohort 3, *n* = 30) expressed lower amount of miR-126 and of miR-218 than pure DCIS (Cohort 2, *n* = 17) (Figure 1C).

### miR-126 and miR-218 inhibit epithelial-to-mesenchymal transition and invasion in DCIS-derived cells

**Figure 2:**
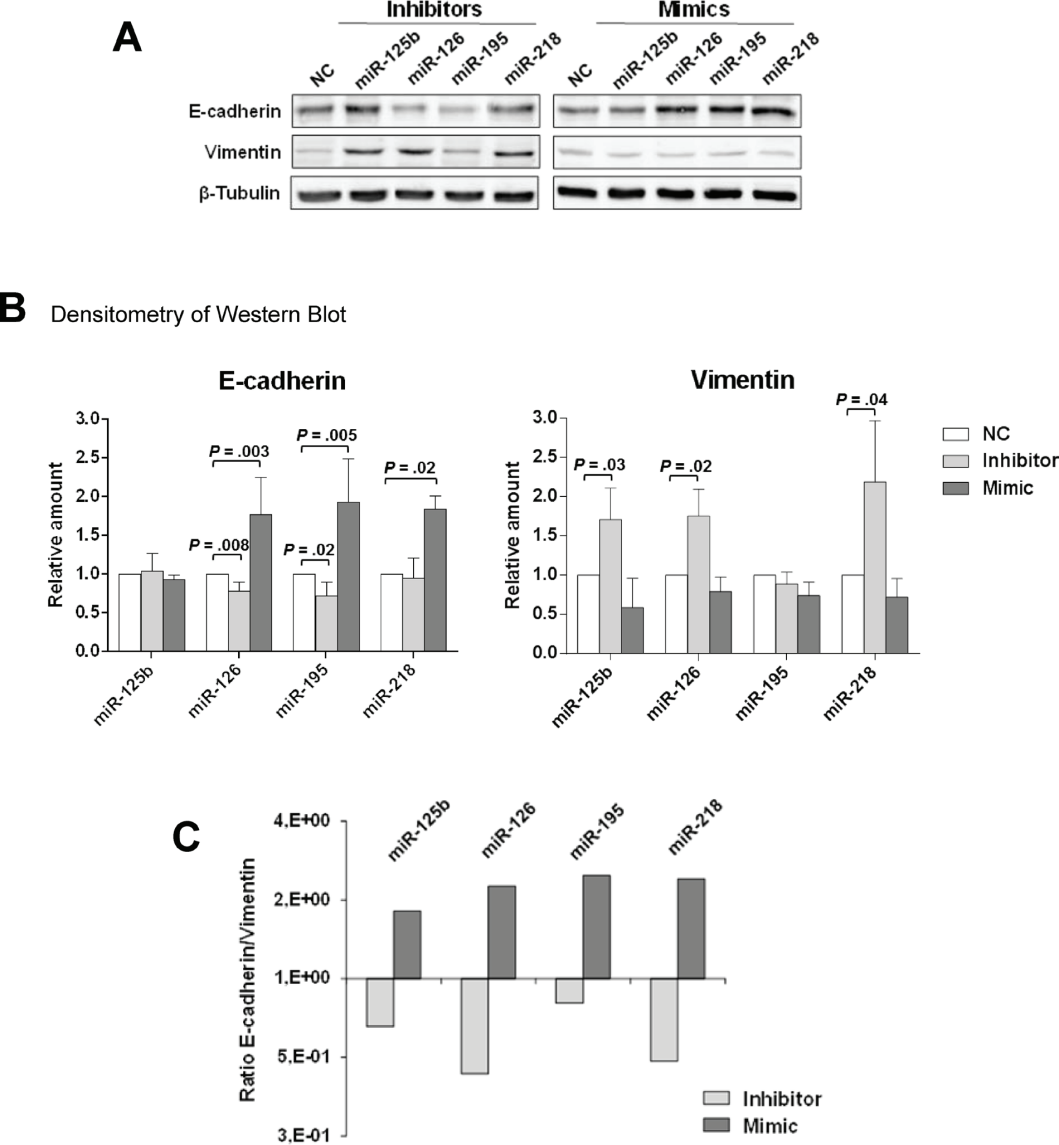
miR-126 and miR-218 inhibit the expression of EMT markers in MCF10DCIS cells (**A**) Representative Western blot analysis of total lysates from MCF10DCIS cells transfected with miRNA inhibitors and mimics, or with their respective negative controls (NC). Random sequences were used as negative controls (NC) and to rule out any contribution from miRNAs in serum. Immune revelation was performed with anti-E-cadherin and anti-Vimentin antibodies. β-Tubulin was blotted as a control of loaded proteins. (**B**) Relative levels of E-cadherin and Vimentin, normalized on β-Tubulin, as determined by densitometry after Western blot. The mean expression level of three separate experiments ± SD is shown. (**C**) Bar plot of the E-cadherin/Vimentin ratio, as measured in B. The relative predominance of E-cadherin over Vimentin upon miRNA treatment established the role of miR-126 and miR-218 in the inhibition of the epithelial-to-mesenchymal transition.

The ability of miR-126 and of miR-218 to regulate markers of the epithelial-to-mesenchymal transition (EMT) was evaluated in MCF10DCIS cells upon treatment with either specific mimics or inhibitors. Two miRNAs often lost in breast cancer, miR-125b and miR-195 [16], were also included in the assay. The levels of the epithelial marker E-cadherin were significantly reduced (*P* = 0.008) by miR-126 inhibitor and were increased by the over-expression of either miR-126 (*P* = 0.003) or miR-218 (*P* = 0.02). Concurrently, the inhibition of the two miRNAs induced a significant increase (*P* = 0.02, *P* = 0.04, respectively) in the mesenchymal marker Vimentin (Figure 2A, 2B). As visualized by plotting the E-cadherin/Vimentin ratio, high levels of miR-126 and miR-218 led to the prevalent expression of the epithelial marker while their inhibition promoted the predominance of the mesenchymal marker (Figure 2C).

**Figure 3:**
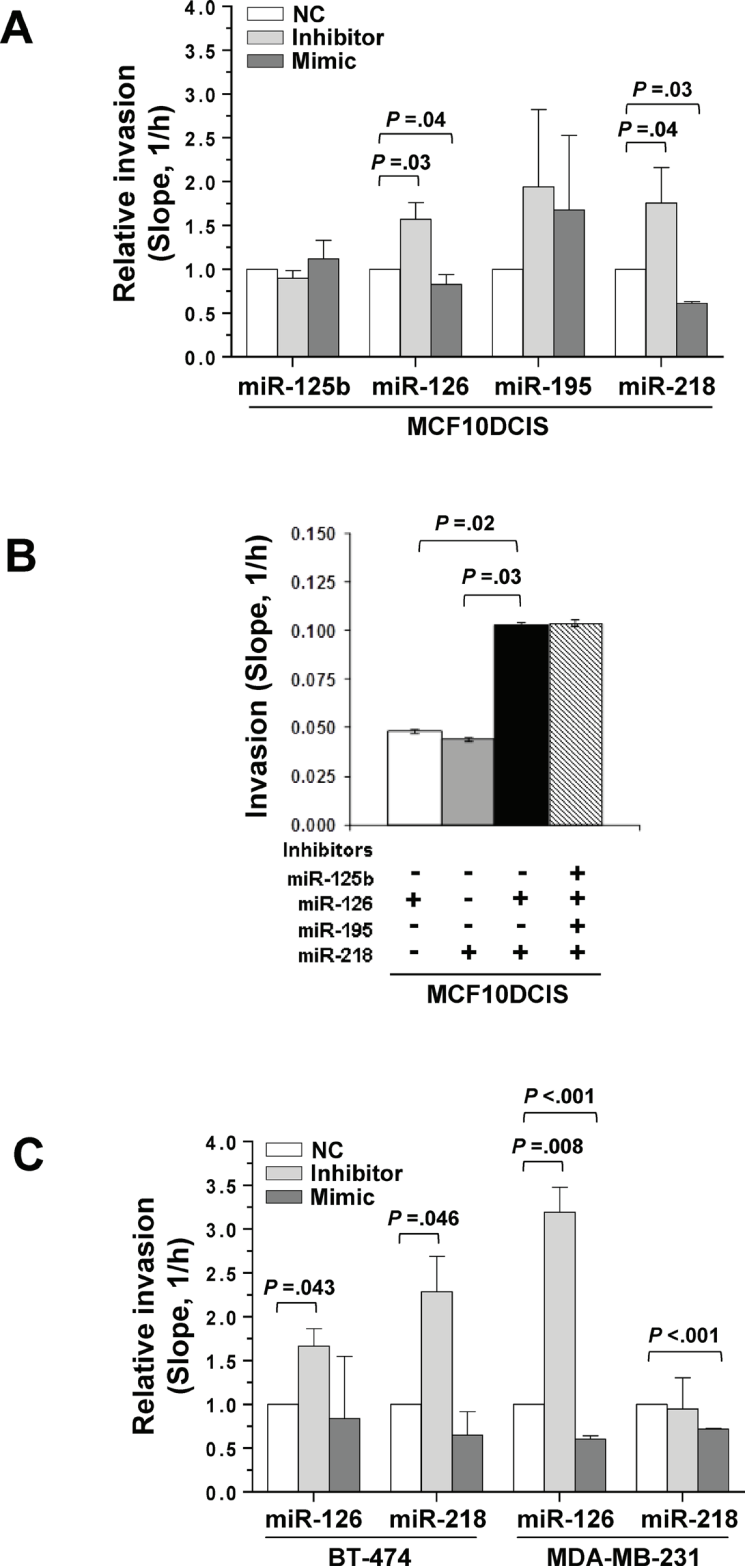
miR-126 and miR-218 inhibit invasion in DCIS and IDC cell lines (**A**) xCELLigence-driven dynamic monitoring of invasion through Matrigel of MCF10DCIS cells transfected with miRNA mimics or inhibitors, or with negative controls (random sequences used as to baseline contributions from miRNAs in serum.). The slope analysis of invasion, that describes the steepness, incline, gradient, and changing rate of the Cell Index curves over time, is shown. In each experimental condition, fold changes in the slope compared with NC is reported. (**B**) Slope analysis of cell invasion after treatment with miRNA inhibitors, single or in combination. (**C**) BT-474 and MDA-MB-231 cells were transfected with miRNA mimics or inhibitors, or with negative controls (NC), and subjected to dynamic monitoring of invasion through Matrigel. In each experimental condition, fold changes in the slope compared with NC is reported. The results are shown as the mean of three separate experiments ± SD.

Since E-cadherin is a major component in the motility circuit activated during EMT [19], the impact of miR-126 and miR-218 on motility and invasion was determined in DCIS-derived cells treated with mimics or inhibitors. xCELLigence real-time analysis on MCF10DCIS cells in which each miRNA was modulated showed that the inhibitors of miR-126 or miR-218 increased the invasion rate through Matrigel (*P* = 0.03, *P* = 0.04, respectively) while their mimics had the opposite effect (*P* = 0.04, *P* = 0.03, respectively) (Figure 3A, Supplementary Figure 2B). The effects of miR-126 and miR-218 were independent, since the invasion scores in MCF10DCIS treated with the miRNAs inhibitors were additive. Under the same conditions, we failed to detect any significant change in the cell proliferation index (Supplementary Figure 2A) as well as any effect on cell cycle (data not shown). Finally, invasiveness was not influenced by co-treatment with inhibitors of other down-regulated miRNAs in IDC such as miR-125b or miR-195 (Figure 3B).

The effects of miR-126 and miR-218 on cell invasion were evaluated in the low invasive BT-474 and in the highly invasive MDA-MB-231 cells, demonstrating that inhibition of each of the two miRNAs increased the ability of both cell lines to pass through Matrigel, while the use of mimics significantly reduced the invasiveness of the MDA-MB-231 cells (Figure 3C).

### Clinical significance of miR-126 and miR-218 on the outcome of DCIS and IDC patients

**Figure 4:**
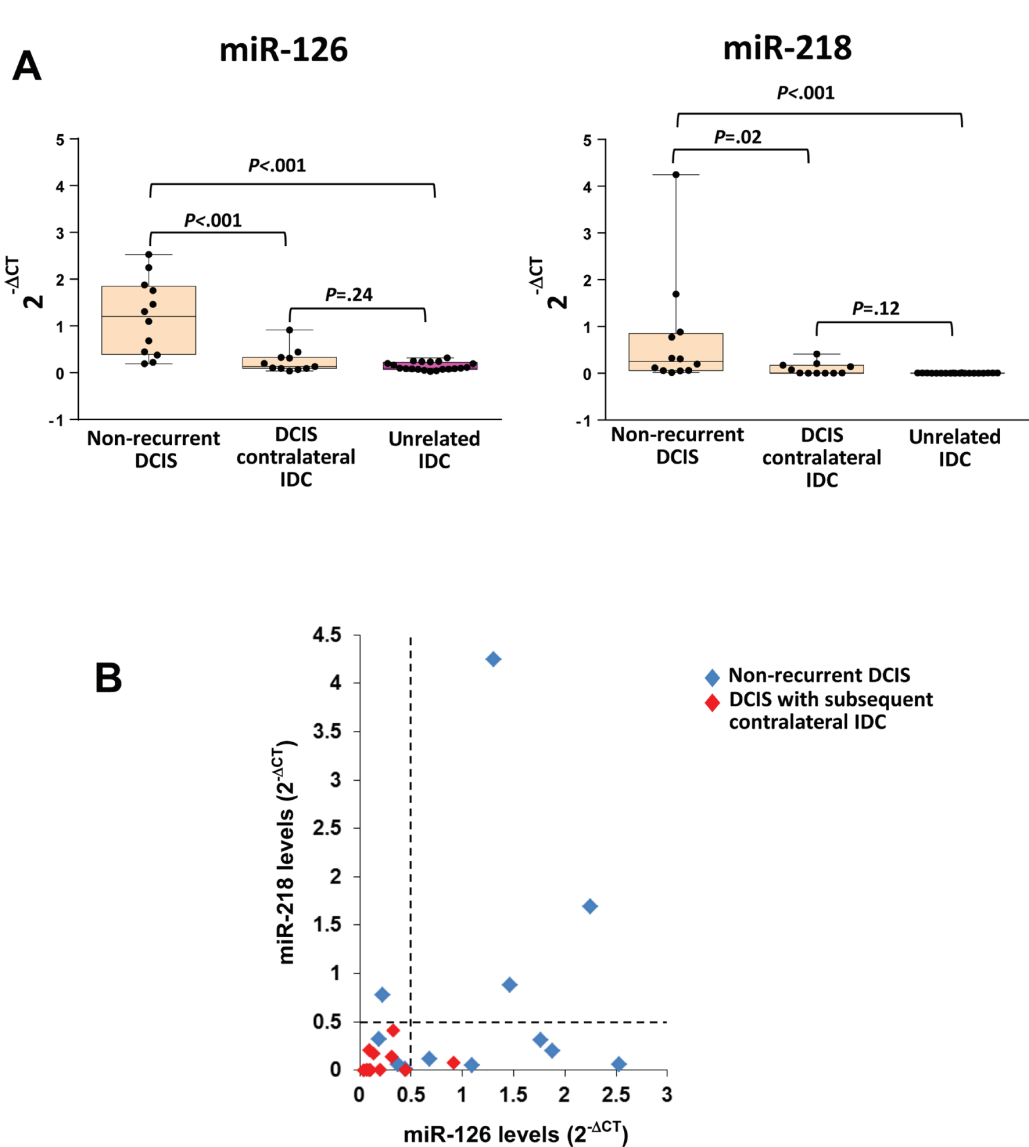
Low miR-126 and miR-218 levels in primary pure DCIS correlate with the contralateral development of IDC (**A**) Box plots showing the expression of miR-126 and mir-218 in DCIS from patients without recurrence (Cohort 4, *n* = 12), in patients who developed a subsequent contralateral IDC (Cohort 4, *n* = 11) and in unrelated IDC (*n* = 20). (**B**) Levels of the two miRNAs in the same DCIS from Cohort 4 patients, in which the dotted lines define arbitrary cut-off between high and low levels of miRNA expression. miRNA levels were determined using the 2^-ΔCt^ method after normalization to U6 snRNA.

To evaluate the prognostic potential of miR-126 and miR-218 we investigated their expression in the DCIS from a set of rare but highly relevant BC patients (Cohort 4, *n* = 23): those that subsequently to DCIS developed a contralateral IDC, i.e. the later appearance of an invasive carcinoma in the other breast. This particular set up was chosen to define a cohort enriched in high-risk DCIS patients. We studied micro-dissected primary unilateral pure DCISs from patients who developed a subsequent contralateral IDC (“high-risk”, *n* = 11), pure DCIS from patients with no recurrence (“low-risk”, *n* = 12), and unrelated IDC (*n* = 20). Again, the levels of miR-126 and miR-218 were significantly higher (*P* = 0.002, *P* = 0.0012, respectively) in indolent DCIS (Figure 4A). The levels of both miRNAs in the high-risk DCIS were similar to those of the IDC. When miR-126 and miR-218 were compared in the same sample, almost all high-risk DCIS expressed low levels of both miRNAs (Figure 4B).

To further investigate the clinical relevance of miR-126 and miR-218 in BC, we measured their association with prognosis in three well-characterized IDC cohorts: TCGA (*n* = 918), METABRIC (*n* = 796) [15], and UK cohort from Oxford (*n* = 210) [21]. miR-218 was positively associated with longer RFS or OS in all three IDC cohorts (OS METABRIC [95% CI, 0.453–0.770 RR], *P<* 0.001; RFS TCGA [95% CI, 0.328–0.938 RR], *P* = 0.03; RFS UK [95% CI, 0.616–0.965 RR], *P* = 0.02), while miR-126 only in one (OS METABRIC [95% CI, 0.581–0.977 RR], *P* = 0.03) (Table 1). The Kaplan-Meier curves showed that patients with higher miR-218 levels had longer relapse-free survival in the TCGA (*P* = 0.03) and UK (*P* = 0.02) cohorts and longer overall survival in METABRIC (*P <* 0.001) (Supplementary Figure 3). As a positive control for the procedure, miR-210 was confirmed as indicator of poor prognosis in all cohorts (Table 1).

**Table 1:** Cox regression analysis of the Overall Survival (OS) and Relapse Free Survival (RFS) of patients enrolled in the three most represented IDC cohorts, TCGA (sequencing), METABRIC (microarray) and UK (microarray)

miRNA	OS TCGA (*n* = 918)		OS METABRIC (*n* = 796)		RFS TCGA (*n* = 650)		RFS UK (*n* = 210)	
	*P*-values	Relative Risk (95% CI)	*P* -values	Relative Risk (95% CI)	*P* -values	Relative Risk (95% CI)	*P* -values	Relative Risk (95% CI)
**hsa-miR-126**	0.07	1.215 (0.985–1.499)	**0.03**	**0.7531** **(0.581–0.977)**	0.44	0.818 (0.493–1.358)	0.32	0.798 (0.512–1.242)
**hsa-miR-218**	0.43	0.921 (0.751–1.130)	**<0.001**	**0.590** **(0.453–0.770)**	**0.03**	**0.555** **(0.328–0.938)**	**0.02**	**0.771** **(0.616–0.965)**
**hsa-miR-210**	**0.04**	**1.249** **(1.009–1.545)**	**<0.001**	**1.621** **(1.246–2.110)**	**0.03**	**1.817** **(1.062–3.111)**	**<0.001**	**2.486** **(1.554–3.977)**

### Cellular roles of miR-126 and miR-218 in breast cancer

The protein coding genes correlated with miR-126 and miR-218 in BC were identified using Spearman correlation, and cross-validation in METABRIC, TCGA, and UK breast cancer cohorts. The use of independent cohorts, each assayed using different technical platforms, ranging from microarrays to RNAseq, ensured a very robust assay by removing major technical and platform related biases. Successively, we performed a Gene Ontology analysis on the correlated coding genes (Supplementary File). High miR-126 and miR-218 were associated with cell adhesion, with miR-218 impacting on the Wnt, Notch, cadherin and integrin signaling pathways. Lack of miR-126 or miR-218, on the other hand was associated with cell proliferation and mitosis.

## DISCUSSION

Once DCIS is detected, the lesion is excised and therapy administered according to the tumor phenotype [2, 5]. Although several randomized trials have confirmed a >50% reduction in the risk of local recurrence with the administration of radiation therapy (RT) compared with breast-conserving surgery alone, controversy persists regarding whether or not RT is needed in “low-risk” patients [7, 8]. Since current approaches do not allow accurate risk stratification, low-risk patients are especially poorly defined in terms of need for adjuvant therapies, which can be associated with short-term and/or long-term effects affecting all-cause mortality. Thus, molecular prognosticators for the risk assessment of DCIS patients are highly sought-after. The diverse roles of miRNA have been extensively studied in breast cancer, but only very few investigations were performed on non-invasive lesions [12, 17, 18]. Here, by using next-generation sequencing, we identified miR-126 and miR-218 as over-expressed miRNAs in pure DCIS when compared to IDC and normal breast. We validated our results in two independent DCIS cohorts [12, 17], confirming that these two miRNAs constitute a signature of non-invasive breast tumors. Most importantly, we detected high miR-126 and miR-218 in pure DCIS with no concomitant invasive lesions, but not in DCIS with adjacent IDC. Finally, lower or no expression of these two miRNAs was also measured in primary pure DCIS from patients who went to develop a contralateral IDC later in life. Thus, our findings, although obtained from relatively small DCIS cohorts, strongly indicate that high levels of miR-126 and miR-218 at diagnosis characterize low-risk DCIS.

The presence of miR-126 and miR-218 was also associated with better prognosis in three large cohorts of patients with invasive breast cancer, reiterating that these miRNAs down-modulate malignant properties, as shown *in vitro* for breast and other solid cancers [20, 21]. In particular, miR-218 was correlated with longer OS or RFS in all cohorts. These results are in agreement with the Gene Ontology analysis showing that miR-126 and miR-218 are involved in cellular adhesion and differentiation, and with previously reported data demonstrating the role of miR-126 in reducing metastasis of breast cancer [22]. At variance with invasive breast tumor cell, in which down-regulation of the two miRNAs is related to cell cycle and mitosis, we cannot correlate their levels with proliferation of non-invasive breast tumors and breast tumor derived cells.

Thus, we propose that the increase of miR-126 and miR-218 in DCIS is part of the mechanism activated by non-invasive tumor cells to counteract tumorigenesis and that the failure of this process, resulting in the down-modulation of these two miRNAs, may have a role in facilitating the progression of DCIS to invasive lesions. Modification of methylation levels, related to expression of miR-126 and miR-218 in mesothelioma [23] and gastric cancer [24], respectively, may be at the basis of this phenomenon.

We tested and validated this hypothesis *in vitro*, using MCF10DCIS cells, the only established DCIS model for the study of the malignant evolution of non-invasive breast cancer [25, 26]. In addition to MCF10DCIS we also studied the BT-474 cell line, derived from a low invasive primary tumor [27] and the highly invasive MDA-MB-231 cells, in which the tumor suppressor role of mir-126 and miR-218 was already demonstrated [21, 28]. Using these models we revealed that miR-126 and miR-218 individually affect the invasion potential of these different cell lines. Additionally, the miRNAs’ action on MCF10DCIS was cumulative, adding to the evidence that their absence in DCIS with invasive propensity was simultaneous.

The EMT program broadly regulates invasion and metastasis and it is assumed to play an important role in the progression of *in situ* to invasive breast carcinoma [19, 29]. The best-characterized alteration in invasion involves the loss by carcinoma cells of E-cadherin, a key cell-to-cell adhesion molecule [19]. Thus we investigated miR-126 or miR-218 for their ability to affect this process in non-invasive BC cells. The inhibition of either miRNA lowered the expression of E-cadherin and, concurrently, up-regulated Vimentin, indicative of a shift from an epithelial to a mesenchymal phenotype.

Both *in vitro* assays and Gene Ontology analysis of patient samples highlighted the role of miR-126 and miR-218 in invasion. Our results are in agreement with those by Lesurf *et al*. [12], who recently reported that biological processes that distinguish DCIS from IDC are related to the microenvironment. They showed that the different subtypes share common features, such as cell adhesion, collagen fibril organization, and ECM-cell receptor interactions. They identified, within each subtype, some DCIS that clustered with IDC in numbers roughly concordant with estimates of indolent/aggressive frequency in DCIS. Nevertheless, they noted that their investigation was not designed to study prognosis due to small sample size and selection of cases.

Our approach was designed in two stages to identify miRNAs related to cancer progression. First, we identified and validated differentially expressed miRNAs in DCIS compared to IDC, using a patient number larger than previously reported in literature, and then we assessed the expression of those miRNAs in two special cohorts of DCIS patients, one with adjacent IDC and the other with subsequent contralateral IDC. Both cohorts included progression events, either local or delayed and distant, thus encompassing divergent but high-risk types of DCIS patients.

In our present work, we aimed to overcome a common limitation in the miRNA studies on DCIS: the very limited size of the cohorts. First, we started with an initial RNAseq cohort of 30 DCIS samples, which is up to now the largest published study on miRNA. Second, in order to further strengthen our findings, we used additional DCIS samples with invasive potential to validate our initial miRNA signature. Thus, instead of looking at miRNAs specific for BC subtypes, our redundant approach allowed us to pinpoint those miRNAs that had a robust pattern of expression across multiple DCIS cohorts with high invasive features. Although we took special care to address the study of small cohorts as outlined above, our conclusions could still be affected by cohorts’ size. Additionally, while we looked for common mechanisms in DCIS, the genotype background and pattern of somatic mutations in DCIS were not investigated. Finally, although we extended the cellular studies to invasive cells, yielding significant results on miRNA roles, repeating the assays on more non-invasive cell lines could lead to further significant results.

Over-diagnosis is a recognized major problem in breast cancer and the lack of prognosticators for early lesions hampers the management of patients. miR-126 and miR-218 are highly expressed in DCIS with low-invasive potential and thus represent important novel biomarkers for the risk assessment in women with early detection of breast cancer. In addition, up-modulation of these two miRNAs could constitute the basis of clinical trials aimed to help to make decisions on therapeutic intervention in low-risk DCIS patients.

## MATERIALS AND METHODS

### Breast tumor tissues

We used four different cohorts of DCIS patients, two with pure DCIS lesions (Cohorts 1 and 2), the third with adjacent DCIS and IDC lesions, and the fourth one with pure DCIS followed or not by the occurrence of IDC in the contralateral breast during follow-up. 30 formalin fixed paraffin-embedded (FFPE) samples of primary pure DCIS (>70% tumor tissue) were selected from a collection of tumors archived at three independent sites: Verona, Padua and Rovigo (Cohort 1) and 17 FFPE breast tissue sections with pure DCIS were from Pisa Archive (Cohort 2). 30 FFPE breast tissue sections with adjacent DCIS and IDC lesions were obtained again from the Pisa archive (Cohort 3). Finally, 20 FFPE samples of primary unilateral pure DCIS from patients with available follow-up data were from the Verona site and 3 from the Rome archive (Cohort 4, *n* = 23). The Cohort 4 included 11 patients who developed a contralateral IDC and 12 control patients who did not recur. The control was selected on the basis of patients’ homogeneity in terms of age (median: 53, range: 51–55), adjuvant therapy and radiotherapy administration. The median follow-up for Cohort 4 was of 94 months (range: 61–120 months). Twenty unrelated IDC samples were used as invasive controls. No samples were excluded from the cohorts.

Written informed consent was obtained from all patients for their tissue samples to be archived and used for research purposes, according to the Helsinki declaration of 1975.

### miRNA-Seq analysis of formalin fixed pure DCIS tissues

The miRNA expression profiles of DCIS Cohort 1 were generated using next-generation sequencing (NGS) on the SOLiD platform (ABI) as described in the Supplemental Materials and were compared with those published by Farazi *et al*. [17], and with the IDCs from The Cancer Genome Atlas (TCGA, cancergenome.nih.gov). Successively, we used for validation the raw data for DCIS and IDC in the Norway study by Lesurf *et al*. [12].

### Laser-capture micro-dissection and miRNA analysis

Samples with pure DCIS (Cohort 2), with adjacent DCIS and IDC (Cohort 3), and with pure DCIS samples from patients with follow-up (Cohort 4), as well the IDC control samples, were laser micro-dissected (LMD) by using the PALM MicroBeam laser microdissector (Carl Zeiss) on 2 μm thick sections. For each sample, 200.000–600.000 μm^2^ were selected and micro-dissected samples were loaded on Maxwell 16 (Promega) to extract RNA, following the Maxwell 16 LEV RNA FFPE protocol. RNA was reverse-transcribed for miR-126 and miR-218 using the TaqMan MicroRNA RT kit (Applied Biosystems). cDNAs in Cohort 2 and 3 were pre-amplified using TaqMan PreAmp protocol (Applied Biosystems) and subsequently droplet digital PCR performed using the QX100™ System (Bio-Rad) according to the manufacturer's instructions. QuantaSoft (Bio-Rad) was used to convert the data into concentrations using Poisson distribution. The miRNAs in DCIS samples from Cohort 4 were quantified by Real-Time qPCR using TaqMan MicroRNA assays (Life Technologies) and a Bio-Rad CFX96™ system. Statistical analysis was performed using the non-parametric median test or Mann-Whitney *U* test for independent samples (SPSS version 21). Two-sided tests were always used and *P* values ≤ .05 were considered statistically significant.

### *In vitro* miRNA activity assays

The human breast cancer-derived cell line MCF10DCIS, one of the very few established models of DCIS [24], was kindly provided by Dr. Macpherson (Beatson Institute for Cancer Research, Glasgow, UK). Cells were grown in Dulbecco's modified Eagle's medium and Ham's F-12 medium (1:1, v/v) (DMEM F-12, Gibco Laboratories) supplemented with 5% horse serum and 1% penicillin-streptomycin (Gibco Laboratories) at 37° C in 5% CO_2_. The BT-474 breast cancer-derived cell line was from ICLC (Genova, I) and was maintained in RPMI 1640 growth medium (Gibco) supplemented with 10% FBS, 1 mM Na pyruvate and 0.01 mg/ml bovine insulin. MDA-MB-231 cells were from the American Type Culture Collection (ATCC) and were maintained in Dulbecco's modified Eagle's medium (DMEM, Gibco Laboratories) supplemented with 10% FBS.

All cell lines were monthly tested for mycoplasm and other contaminations and quarterly subjected to cell identification by single-nucleotide polymorphism.

For specific modulation of miRNAs, transient transfections were carried out with synthetic inhibitors or mimics for miRNAs (miRVana miRNA, Life Technologies) and Lipofectamine 2000 in Opti-MEM I medium (Gibco) without serum. Random sequences were used as negative controls and to check for any contribution from miRNAs in serum. The transfected cells were incubated at 37° C for 48 hours prior to immunochemical and cellular assays.

Total cells lysates (25 μg protein) were separated on 7.5% polyacrylamide SDS gels and blotted to nitrocellulose membranes (GE Healthcare Life Science). The membranes were incubated with antibodies directed against Vimentin and β-tubulin (Sigma) and E-cadherin (Santa Cruz Biotechology), and revealed with ECL (PerkinElmer). The chemiluminescent bands were detected on an ImageQuant™ LAS 4000 imager and densitometry was performed with Image Quant TL (GE Healthcare). Statistical analysis of Western blot data was performed using the non-parametric median test or Mann–Whitney *U* test for independent samples. Two-sided tests were always used and *P* values ≤ 0.05 were considered statistically significant.

Cell proliferation and invasiveness were evaluated with the xCELLigence Real-Time Cell Analyzer System (Roche) that monitors cell events by measuring electrical impedance, as previously reported [30]. To measure cell proliferation, 2500 cells/well were plated and signal detection enabled every 15 min up to 96 hours. For the invasion assay, 4000 cells/well were seeded onto the top chambers covered with a layer of Matrigel (BD Biosciences) diluted 1:20. The bottom chambers were filled with medium containing 10% serum and the signal was detected every 15 min for a total of 24 hours.

### miRNA expression and outcome in ductal carcinoma

The data from the METABRIC study of Dvinge *et al*. [15], accession number EGAS00000000122, were obtained from the European Genome-phenome Archive (EGA), and the normalized miRNA profiles (accession number EGAD00010000438) were studied in relation with overall survival (OS, *n* = 796). The TCGA miRNA profiles for primary BCs were obtained from TCGA data portal (OS, *n* = 918). The data from the Buffa's study [31] (UK cohort, *n* = 210) were obtained from the GEO repository (GSE22216). All miRNA profiles were quantile normalized. The association between continuous miRNA expression and survival times was carried out using univariable Cox regression. The Kaplan–Meier survival curves with the cases divided by miRNA expression (median cut) were constructed for survival times up to 120 months and the log-rank test was used to assess their significance. The analyses were performed using BRB-Array Tools-R/BioConductor (version 4.4) and SPSS (version 21). No samples were excluded from the cohorts.

To pinpoint the cellular roles of miR-126 and miR-218 in breast cancer, we performed a Gene Ontology analysis of the correlated gene sets using the Panther Classification System (as described in Supplementary Materials).

## SUPPLEMENTARY MATERIALS FIGURES AND TABLES





## Abbreviations

DCIS: Ductal carcinoma *in situ*
IDC: Invasive ductal carcinoma
MiRNAs: microRNAs
NGS: next-generation sequencing
EMT: epithelial-to-mesenchymal transition
FFPE: Formalin fixed paraffin-embedded
DMEM F-12: Dulbecco's modified Eagle's and Ham's F-12 media
PBS: Phosphate buffered-saline
