# Supplementary material for: Levels of miR-126 and miR-218 are elevated in ductal carcinoma in situ (DCIS) and inhibit malignant potential of DCIS derived cells
Source: Oncotarget. 2018 May 4;9(34):23543–53. doi: 10.18632/oncotarget.25261 (PMC5955110; doi:10.18632/oncotarget.25261)
Supplement: Supplementary file 1 [file oncotarget-09-23543-s001.pdf]

Levels of miR-126 and miR-218 are elevated in ductal carcinoma *in situ* (DCIS) and inhibit malignant potential of DCIS derived cells

SUPPLEMENTARY MATERIALS

Next-generation sequencing of miRNA

Four consecutive 10 μm thick sections of each pure DCIS sample (Cohort 1) were macro-dissected and subjected to DNA/RNA extraction using AllPrep DNA/RNA FFPE Kit (Qiagen), following the manufacturer's instructions. DNA and RNA were assayed using NanoDrop and Bioanalyzer, respectively. The short RNA was profiled using an RNAseq approach on SOLiD (Applied Biosystems). Briefly, 1 μg of total RNA for each patient was size-selected on Flashpage Fractionator (Ambion). The resulting RNA products were all under 40 nucleotides, as confirmed using the Bioanalyzer 2100 Small RNA chip (Agilent). The total RNA-Seq Kit (Applied Biosystems) was used to produce cDNA libraries as follows: 5′ and 3′ specific adapters were ligated to the RNA, the products were reverse transcribed, purified, and ran on a 6% TBE-Urea gel (Invitrogen). cDNAs from gel slices between 60 and 80 nt in length were excised and used as templates in a 17-cycle PCR reaction with primers specific to the adapter sequences and including a unique barcode for each patient. The PCR reactions were purified and the 110-130 bp fragments were selected on a 6% TBE gel (Invitrogen). The cDNA libraries were then isolated by shredding the gel with elution in PAGE Elution buffer, and precipitated with 5 M ammonium acetate and isopropanol. Templated sequencing beads were produced via emulsion PCR with an Applied Biosystems' EZ Bead system. The templated sequencing beads were finally sequenced on an Applied Biosystems SOLiD 5500 system. The miRNA-Seq data were quality and adapter trimmed using trim galore (http://www.bioinformatics.babraham.ac.uk/projects/trim_galore) which implements cutadapt (Martin, DOI: http://dx.doi.org/ 10.14806/ej.17.1.200) and FastQC (http://www.bioinformatics. babraham.ac.uk/projects/fastqc). We subsequently mapped onto miRBase v21 using NovoAlign (Novocraft Technologies).

Gene Ontology analysis of miRNA expression

The correlated mRNAs to miR-126 and miR-218 were identified using Spearman correlation in the METABRIC, TCGA, UK and Norway BC cohorts, including only the samples with paired miRNA and mRNA expression. The miRNA-associated mRNAs were selected, when cross-validated in both large cohorts: METABRIC (n = 1302) and TCGA (n = 822), Benjamini-Hochberg (BH) corrected p-value <1e-4, and in at least one of the two smaller remaining cohorts: UK (n = 207) and Norway (n = 43), BH corrected p-value <0.001 and 0.05, respectively. This multi-cohort approach was performed to enable the selection of robustly correlated gene-sets across different studies, technical platforms and breast cancer cohorts. The putative targets were selected as the negatively correlated genes that had matching conserved sites to the miRNA seeds according to TargetScan vers. 7.1 (http://www.targetscan.org). To pinpoint the cellular roles of miR-126 and miR-218 in breast cancer, we performed a Gene Ontology analysis of the correlated gene sets using the Panther Classification System (http://www.pantherdb.org) and Gene Ontology (http://geneontology.org/).

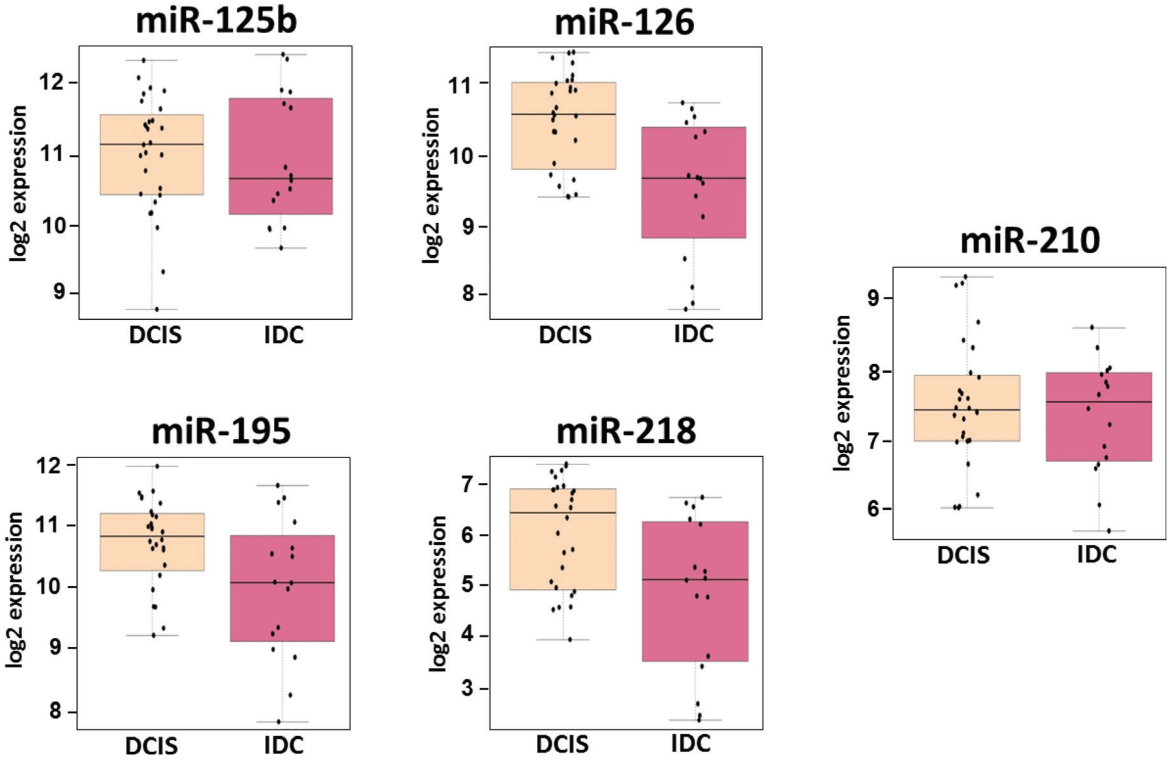

Supplementary Figure 1: The levels of expression for miR-125b, -126, -195, -210 and -218 in DCISs and IDCs of the Norway cohort [12].

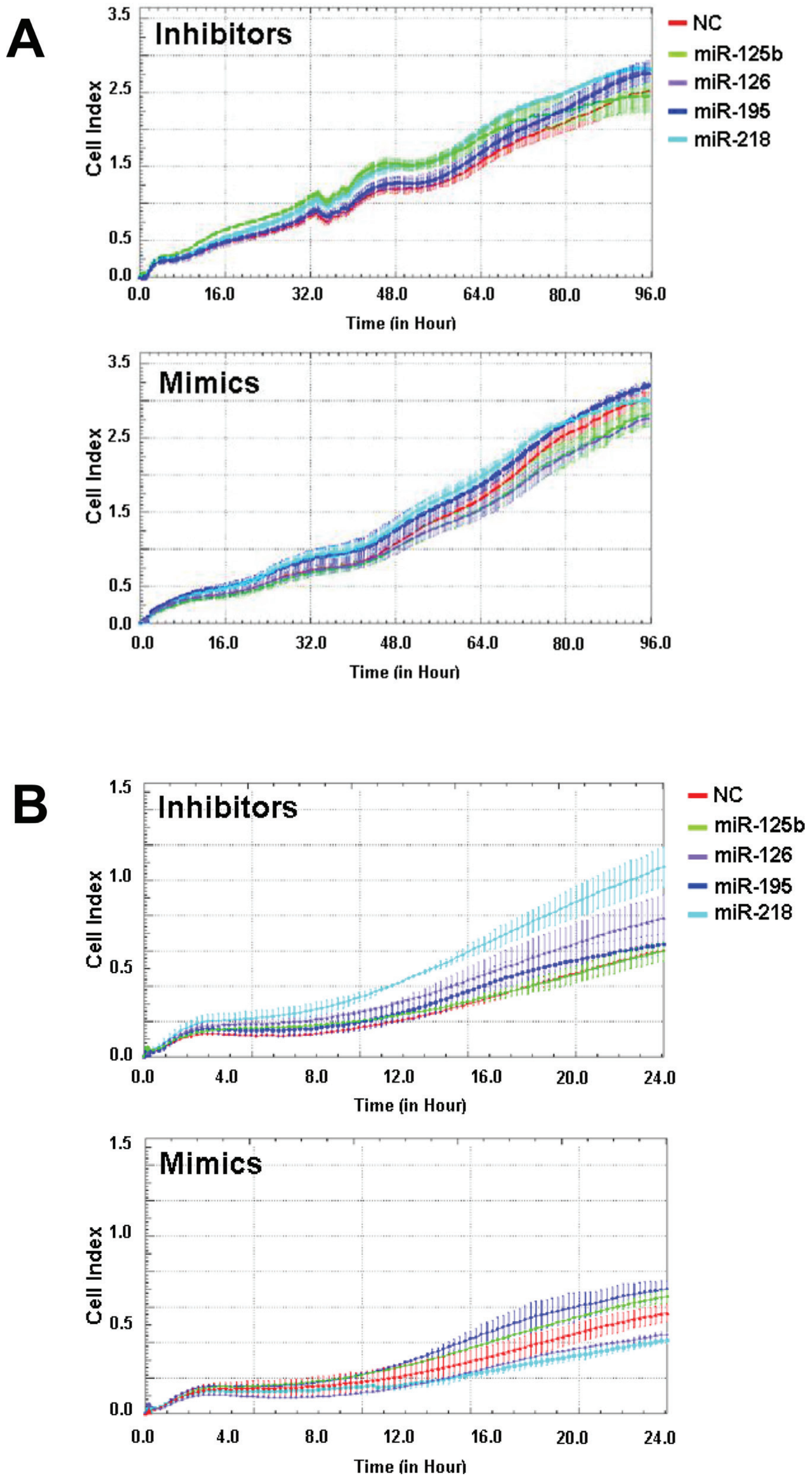

Supplementary Figure 2: miR-126 and miR-218 affect invasion but not proliferation of MCF10DCIS cells. MCF10DCIS cells were transfected with miRNA mimics or inhibitors, or with negative controls (NC), and subjected to dynamic monitoring of proliferation (**A**) or invasion through Matrigel (**B**) using the xCELLigence system. The data are representative of three separate experiments. The mean cell index ± SD is reported. Random sequences were used as negative controls and to balance any contribution from miRNAs in serum.

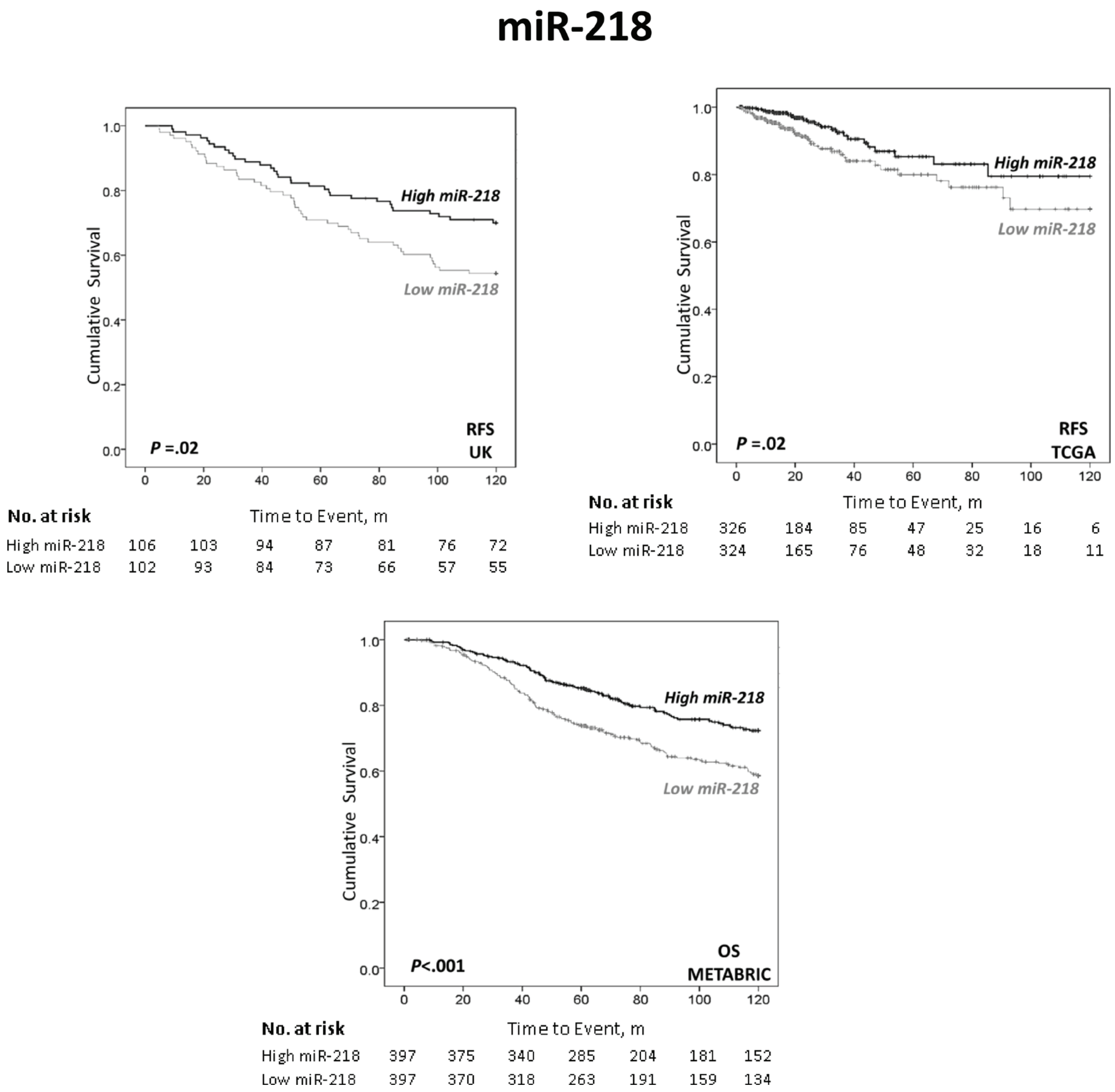

Supplementary Figure 3: miR-218 positively correlates with outcome in three IDC cohorts. The Kaplan Meier curves for miR-218 are shown for each one of three unrelated IDC cohorts. The Log rank test was performed to assess the KM curve significance. The patients were divided in two groups for each cohort according to the median expression level of miR-218. The outcome of patients with higher miR-218 expression is consistently better across the three IDC cohorts.

Supplementary File 1: See Supplementary_File_1
